# microRNA-877-5p exerts tumor-suppressive functions in prostate cancer through repressing transcription of forkhead box M1

**DOI:** 10.1080/21655979.2021.1989969

**Published:** 2021-10-27

**Authors:** Bin Yang, Huifeng Diao, Pu Wang, Fengju Guan, Hechen Liu

**Affiliations:** a Department of Urology, The Affiliated Hospital of Qingdao University, Qingdao, China; b Department of Urology, Heze Municipal Hospital, Heze, China; c Department of Operating Room, The Affiliated Hospital of Qingdao University, Qingdao, China; d Department of Urology, Shandong Provincial Third Hospital, Shandong University, Jinan, China

**Keywords:** Prostate cancer, miR-877-5p, FOXM1, prognosis, proliferation, migration, invasion

## Abstract

The study aimed to investigate the significant potential role of miR-877-5p in Prostate cancer. The expression levels of miR-877-5p and forkhead box M1 (FOXM1) mRNA were detected by qRT-PCR. The prognostic significance of miR-877-5p in prostate cancer was investigated using Kaplan Meier analysis. Then, Cell Counting Kit-8 (CCK-8) and transwell assay were used to evaluate the effects of miR-877-5p on cell biological functions. The mechanism of miR-877-5p action on prostate cancer cells was investigated by luciferase activity assay with wide-type or mutation. miR-877-5p was lowly expressed both in prostate cancer tissues and cell lines compared with corresponding normal counterparts. Further, miR-877-5p was significantly correlated with Gleason score and TNM stage. Moreover, miR-877-5p may serve as an independent prognostic predictor. In addition, FOXM1 was checked as a direct target gene of miR-877-5p, and miR-877-5p can inhibit the expression of FOXM1 to restrain the growth, migration, and invasion abilities of prostate cancer cells. Taken together, miR-877-5p may act as a suppressor in prostate cancer and reduces cancer cell proliferation, migration and invasion by targeting FOXM1. miR-877-5p may serve as the effective biomarkers and therapeutic target for treating prostate cancer patients.

## Introduction

Prostate cancer occurs worldwide, especially frequently happens in middle-aged and elderly men [1,2]. Statistics indicated that prostate cancer is closely related to cancer-related deaths in men, with 248,530 new diagnosed cases and 34,130 cases of death in the United States in 2021 [3–5]. In the last few years, the incidence of prostate cancer in China has also exhibited an increasing trend year by year [6,7]. Obesity, smoking, drinking, and vasectomy are risk factors for prostate cancer [8]. Although the five-year survival rate is high for the localized prostate cancer patients, it still occurs in 28% of patients with distant metastasis [9,10]. Lack of suitable target genes and limited treatment may be the cause of this situation. Thus, it is necessary to seek more effective biomarkers and therapeutic targets for prostate cancer.

MicroRNAs (miRNAs) are a major class of small non-coding RNAs, which is composed of 18 ~ 25 nucleotides and drive gene expression at the messenger RNA (mRNA) level negatively [11–13]. A large number of studies have demonstrated that miRNAs are inseparably connected with the occurrence and progression of prostate cancer, and multiple miRNAs are differentially expressed in nonmalignant tissues and prostate cancer tissues, such as let-7, miR-21, miR-25, and miR-877-5p [14]. There are still many other miRNAs that play important regulatory roles in metastasis that have not yet been discovered. Previous studies have verified that miR-877-5p expression is associated with cervical cancer, liver cancer, and laryngeal cancer, hence, we speculated that whether miR-877-5p plays a crucial role in prostate cancer and clarify whether it can be an independent prognostic factor [15–17].

The relationship between miR-877-5p and FOXM1 were conducted in several studies. For example, miR-877-5p can affect cellular activities by regulating the transcription of forkhead box M1 (FOXM1) in osteoarthritis models [18]. Further, studies have also pointed out miR-877-5p suppresses cell proliferation through FOXM1 in gastric cancer [19]. Based on the above research results, we speculate that miR-877-5p has a close relationship with FOXM1 during the development of cells. whereas, there is no research on the mechanism of miR-877-5p in prostate cancer, let alone combined with FOXM1. Thus, this study was carried out to verify miR-877-5p prognostic correlation, and its influence on the growth and invasion of prostate cancer cells.

## Materials and methods

### Patients and clinical specimens

From June 2012 to May 2015, 101 patients who were pathologically diagnosed as prostate cancer in the Affiliated Hospital of Qingdao University were included in this study. prostate cancer tissue and matching adjacent normal tissue specimens were obtained during surgical resection. After obtaining the sample during the operation, we immediately package the tissue sample, mark and store it. All recruited patients were diagnosed with prostate cancer by at least two histopathologists and had no previous history of anti-tumor treatment. The complete clinical data of patients were collected and recorded (Tables 1 and 2). The grade of prostate cancer tissue and the tumor node metastasis (TNM) stage are determined following the Gleason Grading System and the American Joint Committee on Cancer Classification. After hospitalization, monthly telephone follow-ups were conducted to investigate the survival status of prostate cancer patients and continued statistics for 5 years (Figure 2). The patient or his family members signed an informed consent form on the use of clinical samples and data on a completely voluntary basis. At the same time, the research was endorsed by the Ethics Committee of the Affiliated Hospital of Qingdao University.

**Table 1.** Relationship between miR-877-5p expression and clinical parameters of patients with PCa

Variables	Cases (n = 101)	miR-877-5p expression		*P*-value
		Low (n = 53)	High (n = 48)	
Age				0.940
≤60	53	28	25	
>65	48	25	23	
Tumor size				0.849
≤3 cm	62	33	29	
>3 cm	39	20	19	
PSA				0.196
≤10 ng/ml	65	31	34	
>10 ng/ml	36	22	14	
Surgical margin				0.433
Negative	72	36	36	
Positive	29	17	12	
Prostate volume				0.878
≤50 ml	66	35	31	
>50 ml	35	18	17	
TNM stage				0.011
I–II	54	22	32	
III	47	31	16	
Gleason score				0.047
≤7	68	31	37	
>7	33	22	11	

PSA, prostate-specific antigen; TNM, tumor-node-metastasis; PCa, prostate cancer.

**Table 2.** Multivariate Cox analysis of clinical characteristics in relation to overall survival

Characteristics	Multivariate analysis		
	HR	95% CI	*P*
miR-877-5p	3.880	1.367–11.011	0.011
Age	1.001	0.433–2.313	0.998
Tumor size	1.563	0.644–3.794	0.324
PSA	2.240	0.909–5.520	0.080
Surgical margin	1.006	0.379–2.669	0.990
Prostate volume	1.461	0.623–3.429	0.384
TNM stage	4.778	1.334–17.106	0.016
Gleason score	2.091	1.226–7.161	0.016

### Cell lines and culture

The cell lines used in this experiment including four human prostate cancer cell line (22RV-1, LNCap, PC-3, and DU145) and the normal prostate epithelial cell line (RWPE-1). Among them, RWPE-1, PC-3, and DU 145 cell lines were stored in our laboratory, others were obtained from the Cell Bank of the Chinese Academy of Sciences (Shanghai, China). All cells were cultured in RPMI 1640 medium (Oshima, USA) containing 10% FBS. The cells were placed in an incubator with 5% CO_2_ at 37°C for further culture.

### Cell transfection

Before transfection, prostate cancer cells were seeded in RPMI 1640 medium and incubated overnight. When the cell fusion achieved about 70%-80%, the cells were collected and resuspended in a serum-free culture medium. The miR-877-5p mimic and mimic negative control (mimic NC) were obtained from Guangzhou RIBOBIO (China). Then prostate cancer cells were incubated into the plate, and cell transfection experiments were carried out using the lipofectamine 2000 kit (Invitrogen, USA) [20]. Untreated cells were used as control.

### Prediction of downstream target gene

The biological prediction websites Targetscan (www.targetscan.org) and StarBase (http://starbase.sysu.edu.cn/) were used to predict the targeted binding between miR-877-5p and FOXM1 (ENSG00000111206).

### Quantitative real-time PCR assay

RNAs were isolated from prostate cancer tissues and cultivated cells using RNAzol (Sigma-Aldrich). PrimeScript Reverse Transcriptase Kit (Takara) and miRNA 1st Strand cDNA Synthesis Kit (Vazyme) were used to reverse transcription to synthesize cDNA for transcription factor and miRNA respectively. Subsequently, the qRT-PCR assay was conducted with ChamQ Universal SYBR qPCR Master Mix (Vazyme) and miRNA Universal SYBR qPCR Master Mix (Vazyme) [21]. The expression of miR-877-5p or FOXM1 was detected using the 2^−ΔΔCt^ method normalizing to U6 levels or GAPDH levels, respectively.

### Cell Counting Kit-8 (CCK-8) proliferation assay

The proliferation ability of DU-145 and LNCap cells was assessed by CCK-8 assay (Dojindo) [22]. The transfected cells (5 × 10^3^ cells/well) were seeded in a 96-well cell culture plate and cultured at 37°C. Then 10 μl of CCK-8 reagents (5 mg/ml; Sigma; Merck KGaA) were added at 0, 24, 48, and 72 h, respectively. Subsequently, the optical density value of the cells was measured at 450 nm by a microplate reader. The experiment was measured at least three times at each time point.

### Transwell cell migration and invasion assay

The migratory and invasive ability of prostate cancer cells was measured in a Transwell chamber [23]. Before the start of the invasion test, Matrigel should be applied to the membrane 6 hours in advance. The medium that supplemented 10% FBS was added to the bottom chambers. Then, LNCap and DU-145 cells were added to the upper chambers. After 48 hours, migrated or invaded cells were stained with 0.1% crystal violet (Sigma-Aldrich) in the lower chamber and counted using a microscope.

### Dual-luciferase reporter assay

To acquire the reporter vector FOXM1-wide-type (FOXM1-WT), the sequences of FOXM1 were synthesized and cloned into the pmirGLO vector (Promega, Shanghai, China) [24]. The FOXM1-mutated (FOXM1-MUT) was generated using corresponding FOXM1 3ʹUTR mutant versions without miR-877-5p binding sites. Then DU-145 cells were incubated in 24-well plates and respectively co-transfected with miR-877-5p mimic, mimic NC, miR-877-5p inhibitor, or inhibitor NC with the help of Lipofectamine 2000 (Invitrogen). The luciferase activity was measured after 48 h of transfection.

### Statistical analysis

Statistical analysis of the data was processed using IBM SPSS 20.0 software (SPSS, USA) and GraphPad Prism 7.0 software (GraphPad Software, USA). All experiment results are repeated three or more times. During processing, the chi-square (χ^2^) test was used to compare the relationship between miR-877-5p expression and clinic data of patients. One-way analysis of variance (ANOVA) was performed to estimate the significant differences among multiple groups and paired Student’s t-test was used to compare the statistical significance between two groups. Kaplan-Meier analysis and Cox regression analysis were used to explore whether miR-877-5p has prognostic significance.

## Results

In the current study, we performed a series of experiments to explore the clinical significance and functional role of miR-877-5p in prostate cancer. In addition, the regulatory mechanism of miR-877-5P/FOXM1 in Prostate cancer was tentatively explored. These investigations were carried out to help provide a potential prognostic biomarker and future therapeutic target for Prostate cancer patients.

### miR-877-5p expression in prostate cancer tissues and cell lines

**Figure 1.**
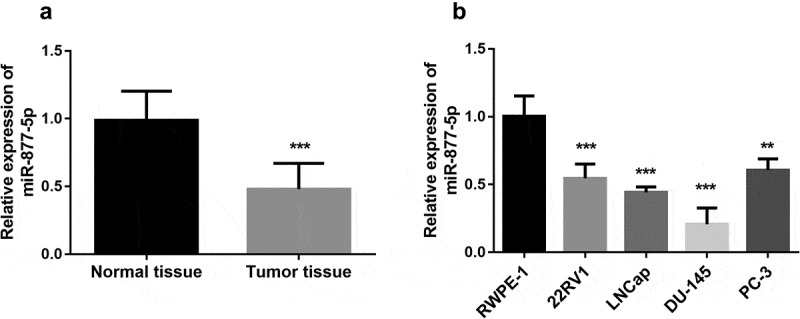
miR-877-5p is downregulated in prostate cancer. (a). Relative expression of miR-877-5p in prostate cancer tissue and normal tissue samples (n = 101). (b). miR-877-5p expression in prostate cancer cell lines and normal prostatic epithelial cell RWPE-1. n = 4. *P < 0.05, ***P < 0.001

The expression of miR-877-5p was measured in paired prostate cancer tissues and adjacent normal tissues. Compared to adjacent normal tissues, miR-877-5p was diminished in tumor tissues (Figure 1(a), *p* < 0.001). Further, the expression of miR-877-5p in different cell lines was measured by qRT-PCR analysis. As presented in Figure 1(b), miR-877-5p was obviously down-regulated in all cell lines compared to the normal prostate epithelial cell line (all *P* < 0.01). According to the results, miR-877-5p expression levels significantly declined in the DU-145 cell line, followed by the LNCap cell line (*P* < 0.001). Since DU-145 and LNCap cell lines have similarly low miR-877-5p expression levels, both cells are used in subsequent experiments.

### The expression of miR-877-5p was related to clinicopathological features in prostate cancer

Besides, the connection between miR-877-5p expression in tissues and clinical features of prostate cancer patients was analyzed. Based on the mean miR-877-5p expression value (0.4803) in tumor tissues, all prostate cancer patients were subdivided into miR-877-5p low expression group (n = 53) and high expression group (n = 48). The χ^2^ test revealed that miR-877-5p expression was associated with the Gleason score (*P* = 0.047) and TNM stage (*P* = 0.011). However, the expression of miR-877-5p was not correlated with other characteristics, such as age, tumor size, prostate volume, surgical margin, and PSA (all *P* > 0.05, Table 1).

### miR-877-5p was associated with poor prognosis in patients with prostate cancer

**Figure 2.**
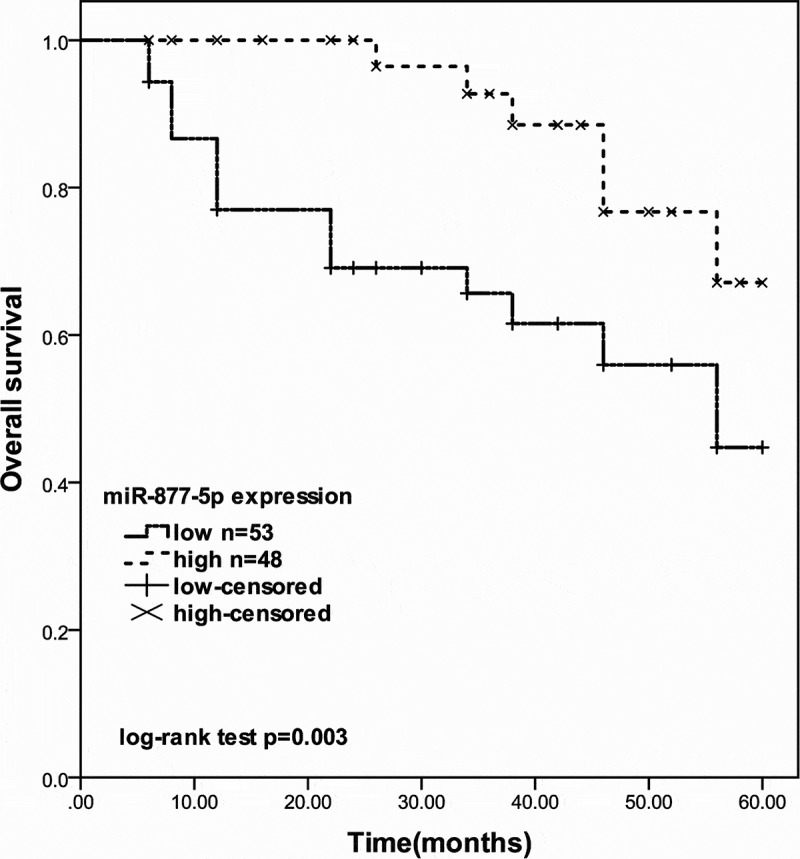
The 5-year survival rate of prostate cancer patients with different miR-877-5p expression levels (log-rank test *P* = 0.003)

According to the Kaplan-Meier curve based on overall survival information of prostate cancer, the 5-year overall survival rates of prostate cancer patients with low expression of miR-877-5p were shorter than the patients with high miR-877-5p expression (log-rank test *P* = 0.003, Figure 2). Furthermore, Multivariate Cox’s regression analysis results indicated that miR-877-5p expression level (*P* = 0.011) is an independent risk prognostic factor in prostate cancer (Table 2).

### miR-877-5p suppressed prostate cancer cellular behaviors

**Figure 3.**
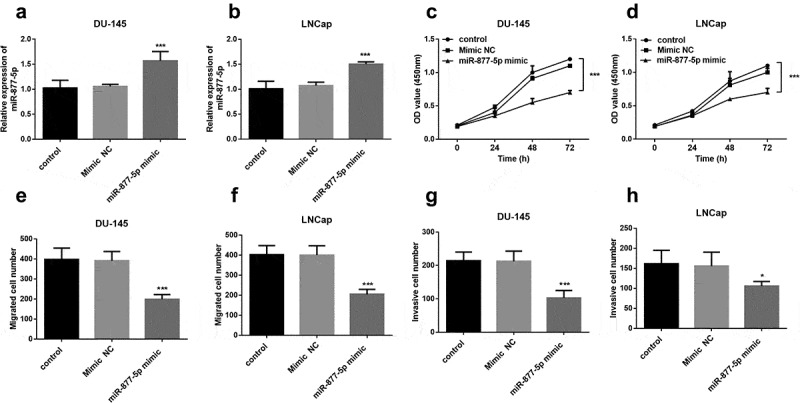
Proliferation of DU-145 and LNCap cells was suppressed after the upregulation of miR-877-5p compared to untreated cells. DU-145 and LNCap cells were transfected with mimic NC or miR-877-5p mimic. (a) and (b) miR-877-5p expression was determined in DU-145 and LNCap cells by qRT-PCR. n = 5. (c) and (d) Proliferative capacity of DU-145 and LNCap cells were measured by CCK-8. n = 3. (e) and (f) Migratory ability of DU-145 and LNCap cells were measured by transwell assay; n = 5. (g) and (h) Invasive ability of DU-145 and LNCap cells were detected using transwell assay. n = 5. *P < 0.05, ***P < 0.001

To determine whether miR-877-5p has a functional role in prostate cancer, the expression of miR-877-5p was upregulated using miR-877-5p mimic that transfected into DU145 and LNCap cells. miR-877-5p expression was significantly augmented by a miR-877-5p mimic in DU145 and LNCap cells (all *P* < 0.001, Figure 3(a,b)). Subsequently, a CCK-8 assay was used to assess cell proliferation capacity. The results revealed that the increased expression of miR-877-5p decreased cell proliferation (*P* < 0.001, Figure 3(c,d)). Also, transwell migration and invasion assay results demonstrated that miR-877-5p mimic caused a drop of the migratory and invasive capacities of DU145 and LNCap cells (all *P* < 0.05, Figure 3(e–h)). Based on the above experiments, we found that there is no difference between the DU145 and LNCap cell lines in characterizing prostate cancer, and miR-877-5p inhibits the progression of prostate cancer cells.

### miR-877-5p played a role in prostate cancer by targeting FOXM1

**Figure 4.**
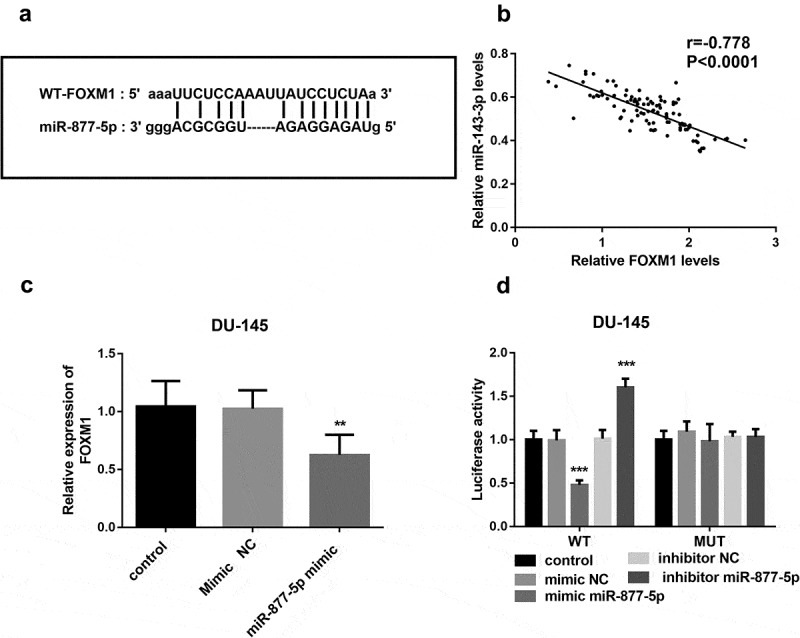
miR-877-5p interacted with FOXM1. (a) The binding site between FOXM1 and miR-877-5p is shown. (b) The correlation between miRNA-143-3p and miR-877-5p expression in prostate cancer tissues was analyzed by Spearman correlation analysis. n = 101. (c) Expression of FOXM1 was measured using qRT-PCR in DU-145 cells that were transfected with mimic NC or miR-877-5p mimic separately; n = 5. (d) Luciferase activity was examined in DU-145 cell cotransfected with miR-877-5p mimic, mimic NC, miR-877-5p inhibitor, or inhibitor NC and WT-FOXM1 or MUT-FOXM1. n = 3. **P < 0.01, ***P < 0.001

To explore whether miR-877-5p and FOXM1 can interact in prostate cancer, we used the DU-145 cell line, in which miR-877-5p has an especially high expression, to predict the relationship between FOXM1 and miR-877-5p. The bioinformatic analysis showed that FOXM1 and miR-877-5p could form multiple bases pairing in (Figure 4(a)). Spearman’s rank correlation coefficient analyzed the correlation between the expression levels of miR-877-5p and FOXM1. The expressions of miR-877-5p and FOXM1 were inversely and significantly correlated with each other (r = −0.778, *P* < 0.001, Figure 4(b)). The FOXM1 expression was downregulated in DU-145 cells with high expression of miR-877-5p (*P* < 0.01, Figure 4(c)). Subsequently, a mutated FOXM1 construct was obtained by mutating the binding sites on m FOXM1. According to the luciferase assay, the luciferase activity was not affected by the expression of miR-877-5p in the MUT transfected cells, while was decreased by miR-877-5p overexpression and increased by the inhibited expression of miR-877-5p in the WT transfected cells (*P* < 0.001, Figure 4(d)). This suggested that miR-877-5p may bind to the predicted sites and then inhibit the expression of FOXM1.

## Discussion

Regarding miRNAs, more and more studies demonstrated that they can transmit signals in cells and regulate gene expression [25]. Besides, miRNAs also play an important role in cancer cells, and maybe tumor suppressors or oncogenes are involved in tumor progression [26,27]. Similarly, in prostate cancer tissue, there are also some miRNAs with ectopic expression [27–29]. Based on the above, this study aimed to study the previously undiscovered new role of miR-877-5p acting as a tumor-inhibitory role in prostate cancer .

According to the results of qRT-PCR experimental data, miR-877-5p expression in both prostate cancer cells and prostate cancer patient tissues was significantly reduced. In subsequent experiments, it is observed that miR-877-5p expression is related to the Gleason score and TNM stage of prostate cancer. These results suggested that miR-877-5p may serve as a tumor suppressor in prostate cancer. Interestingly, a recent study revealed that miR-877-5p was upregulated in advanced castration-resistant prostate cancer with adenocarcinoma characteristics (CRPC-Adeno) compared to castration-resistant prostate cancer to androgen-independent neuroendocrine states (CRPC-NE) [30]. The results suggest that specific subtypes may have different characteristics. Additionally, patients who have low expression of miR-877-5p own a significantly worse prognosis compared with the group with high expression of miR-877-5p on a 5-year follow-up study and miR-877-5p expression might be a prognostic risk predictor for prostate cancer. Combine the above cancers, miR-877-5p may serve as a prognostic biomarker in prostate cancer.

Function experiments results indicated that miR-877-5p mimic suppresses the prostate cancer cell biological activity. A similar finding was observed in the current study, whereby the transfection with the miR-877-5p mimic could decrease proliferation in the gastric cancer cells [19]. The tumor-suppressor role of miR-877-5p was also demonstrated in other types of cancers, such as gastric cancer [31,32] and laryngeal squamous cell carcinoma [33]. Therefore, we inferred that miR-877-5p may restrain prostate cancer cell proliferation, migration, invasion, and involvement in the tumorigenesis and progression of prostate cancer.

Forkhead Box M1 (FOXM1), a member of the Forkhead box family, shares a 100 amino acid long winged-helix DNA-binding domain, which includes four subtypes: FOXM1A, B, C, and D [34,35]. In this article, this study focused on FOXM1B, which is also called FOXM1, which is different from other FOXM1 isomers in that it mainly regulates the growth, migration, and angiogenesis of cancer cells [34,36,37]. A recent study in 18,000 cancer cases involving 39 human malignancies showed FOXM1 regulatory network is an important predictor of poor prognosis [38,39]. This conclusion further confirmed the important role of FOXM1 in prostate cancer.

Recently, it is observed that FOXM1 expression has a potential prognostic value for prostate cancer, however, there is some evidence verifying that miR-877-5p can improve chondrocyte function of experimental osteoarthritis(OA) by targeting FOXM1 as a postulated molecular mechanism [18,39]. Then, the relationship between miR-877-5p and FOXM1 caught our attention, we speculated miR-877-5p may bind to FOXM1 to achieve the results of regulating cell biological function in prostate cancer. There is no doubt that our hypothesis is confirmed because of the bioinformatics prediction result of the targeted binding between miR-877-5p and FOXM1. Further analysis evidenced that FOXM1 and miR-877-5p are negatively related to each other and FOXM1 may be the direct target of miR-877-5p. Thus, our study revealed that miR-877-5p may also drive cell proliferation, migration, and invasion of prostate cancer cells by targeting FOXM1. However, with the consideration of specific subtypes of prostate cancer may have different characteristics, the detailed mechanism of miR-877-5p in prostate cancer need to be further investigated in future in vivo and in vitro studies.

## Conclusion

Our finding in prostate cancer, a down-regulation of miR-877-5p, supports the recent discussion that the miR-877-5p can act as a tumor suppressor microenvironmental factor. The miR-877-5p is downregulated in prostate cancer tissues and cells, and its high expression of miR-877-5p attenuates cancer cell proliferation potential, migration abilities, and invasion capacity by targeting FOXM1. Low miR-877-5p expression may be a novel prognostic marker in prostate cancer. Therefore, our study proved that miR-877-5p has the potency to be further developed as a biomarker and future therapeutic target.
